# A New Multi-Branch Convolutional Neural Network and Feature Map Extraction Method for Traffic Congestion Detection

**DOI:** 10.3390/s24134272

**Published:** 2024-07-01

**Authors:** Shan Jiang, Yuming Feng, Wei Zhang, Xiaofeng Liao, Xiangguang Dai, Babatunde Oluwaseun Onasanya

**Affiliations:** 1 School of Computer Science and Engineering, Chongqing Three Gorges University, Chongqing 404100, China; jiangshan@sanxiau.edu.cn (S.J.); daixiangguang@163.com (X.D.); 2 Key Laboratory of Intelligent Information Processing and Control, Chongqing Three Gorges University, Wanzhou, Chongqing 404100, China; 3 College of Computer Science, Chongqing University, Chongqing 400044, China; xfliao@cqu.edu.cn; 4 Department of Mathematics, University of Ibadan, Ibadan 200005, Nigeria; bo.onasanya@ui.edu.ng

**Keywords:** traffic congestion detection, image data, target detection, feature map, classification model

## Abstract

With the continuous advancement of the economy and technology, the number of cars continues to increase, and the traffic congestion problem on some key roads is becoming increasingly serious. This paper proposes a new vehicle information feature map (VIFM) method and a multi-branch convolutional neural network (MBCNN) model and applies it to the problem of traffic congestion detection based on camera image data. The aim of this study is to build a deep learning model with traffic images as input and congestion detection results as output. It aims to provide a new method for automatic detection of traffic congestion. The deep learning-based method in this article can effectively utilize the existing massive camera network in the transportation system without requiring too much investment in hardware. This study first uses an object detection model to identify vehicles in images. Then, a method for extracting a VIFM is proposed. Finally, a traffic congestion detection model based on MBCNN is constructed. This paper verifies the application effect of this method in the Chinese City Traffic Image Database (CCTRIB). Compared to other convolutional neural networks, other deep learning models, and baseline models, the method proposed in this paper yields superior results. The method in this article obtained an F1 score of 98.61% and an accuracy of 98.62%. Experimental results show that this method effectively solves the problem of traffic congestion detection and provides a powerful tool for traffic management.

## 1. Introduction

As the transportation system continues to grow, the number of motor vehicles continues to rise, resulting in severe traffic congestion in some areas of large cities [1,2]. Efficient and reasonable traffic congestion monitoring helps transportation departments manage congestion problems more effectively [3,4]. Currently, there are three main methods that can be applied to traffic congestion monitoring. The first method is to detect congestion through induction coils, which can effectively measure the traffic flow and speed on the road [5,6]. An induction coil is a device used to measure vehicle speed, commonly used in traffic control and traffic monitoring systems. Its working principle is based on the changes in induced electromagnetic fields. The induction coil consists of a coil, usually buried in the road, with a length perpendicular to the direction of vehicle travel. When a vehicle passes over the speed measurement coil, the metal parts of the vehicle (such as the wheels) will disturb the electromagnetic field generated by the induction coil. This disturbance will cause changes in the induced current within the coil. The induction coil calculates the speed of the vehicle by measuring the changes in induced current [7].

The second method is to use vehicle GPS data for congestion monitoring [8,9]. GPS equipment can provide real-time feedback on vehicle position and speed. The principle of GPS speed measurement is based on the Doppler effect. The Doppler effect refers to the change in the frequency of the received signal when the transmitting source and the receiving source are in relative motion. In GPS speed measurement, after receiving the signal transmitted by the satellite, the receiver measures the frequency of the signal and calculates its own speed relative to the satellite based on the Doppler effect. By measuring and calculating the frequencies of multiple satellite signals, GPS receivers can accurately measure their own speed [10]. By calculating the average speed of vehicles on each road section, it can be determined whether there is congestion on that road section. Although these two methods are relatively simple in detecting congestion, they require extensive hardware support. Installing induction coils on various road sections and installing GPS equipment in vehicles will consume a lot of manpower and financial resources. This article will focus on proposing a traffic congestion detection method with less hardware consumption.

The third method is to use camera data and computer vision algorithms to detect whether there is congestion on the road [11,12]. At present, a large number of cameras have been widely deployed in various sections of transportation system. Using these existing cameras for traffic congestion monitoring can reduce hardware resource requirements, but requires the application of complex algorithms for calculations. Therefore, the focus of this research is to use camera data to determine whether there is congestion in each road section. We will study image recognition models for traffic congestion detection. The input of the model is image data captured by cameras, and the output is whether there is congestion on the road section. Currently, there are three main types of research in this field. (1) The first method uses a target detection algorithm to detect the number and location of cars, trucks, and buses in the picture, and based on this, determine whether there is congestion on the road [13,14]. (2) The second method extracts traffic status features from images, including the number of vehicles and their speed, and then uses machine learning models to determine whether there is congestion in the image based on these features [15,16]. (3). The third method uses the image classification model to determine whether there is congestion in the area where the camera is located [17,18].

The target detection models used for traffic congestion detection mainly consist of the following: the Haar Cascade [15,19], You Only Look Once (YOLO) [20,21], Single-Shot MultiBox Detector (SSD) [22,23], and Mask R-Convolutional Neural Networks (R-CNN) [24,25].

Many scholars have conducted traffic-related image detection and image classification based on visual features. In terms of features, they mainly consist of bag features [26], Haar features [27], edge features [28], shape features [29], gradient histograms features [30] and CNN [31].

Computer vision models used for traffic image classification mainly consist of VGG-16 [32,33], ResNet50 [34,35], EfficientNet [36,37], and Inceptionv3 [38,39].

In the problem of traffic congestion detection based on camera data, current research mainly relies on machine learning methods based on feature extraction or image classification models based on deep learning [9,40]. These methods currently have problems such as insufficient detection accuracy and excessive model size. The research in this article is mainly based on object detection models. This article will propose a new vehicle information feature map (VIFM) for extracting traffic congestion features. Meanwhile, this article will propose a new multi-branch convolutional neural network (MBCNN) for traffic congestion detection.

In building an image-based traffic congestion recognition model, the main challenges include accurate detection of vehicles in images, reasonable feature extraction, and efficient classification model construction. The reason why this article adopts a deep learning-based approach is that deep learning has achieved great success in the image field. We apply deep learning methods to traffic congestion recognition in order to make some progress in the problem of traffic congestion recognition.

This study uses a target detection algorithm to process the images captured by the camera to automatically identify traffic elements such as vehicles in the images. Divide the picture into several small squares, and then count the number of vehicles in each small square. This article extracts the maximum value, sum value and number of small squares greater than the threshold to extract higher-level features. By extracting image features through this method, the model can learn the difference between congestion and non-congestion states, laying the foundation for subsequent congestion detection.

Next, this article proposes a new multi-branch convolutional neural network model. The three branches of the model each use a different size of convolution kernels. Finally, the outputs of the three branches are passed through a fully connected layer to determine whether there is congestion. This classification model is used in this article for traffic congestion prediction detection.

Finally, this study combines actual traffic data to train and test the proposed congestion detection method. In the testing phase, the actual traffic conditions are compared with the detection results to evaluate the F1 score and accuracy of the model. The main contribution of this paper is to propose a new method for extracting vehicle information feature maps (VIFMs) and a multi-branch convolutional neural network model (MBCNN). The model proposed in this article will achieve better results compared to existing convolutional neural networks, deep neural networks, and baseline models. The method proposed in this paper can achieve traffic congestion detection based on camera data. Due to the limitations of our experimental conditions, we can only use cameras to capture 2D images. At the same time, our method can effectively provide intelligent algorithms for massive ordinary traffic surveillance cameras without adding additional hardware costs.

## 2. Method

The traffic congestion detection in this article is mainly divided into three aspects: vehicle target detection, feature map extraction, and classifier detection of whether there is traffic congestion. First, this article establishes a vehicle information extraction model based on the You Only Look Once v8 (YOLOv8) model. Then, this paper proposes a feature map extraction method based on vehicle information. Finally, this article establishes a multi-branch convolutional neural network model for traffic congestion detection.

### 2.1. Target Detection

**Figure 1:**
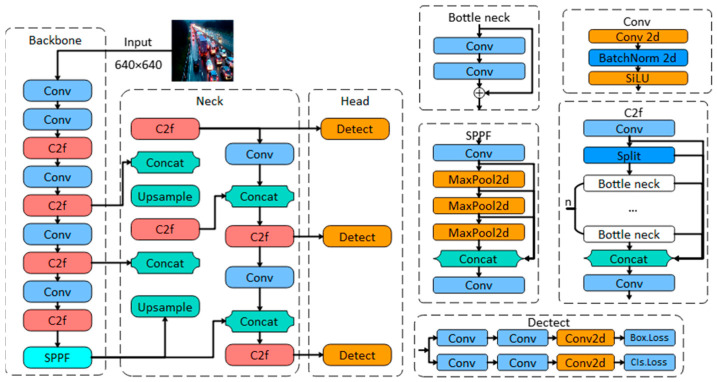
Simplified structure diagram of YOLOv8 model [39].

This article uses the most advanced YOLOv8 target detection model to detect the location and number of vehicles in each picture. The structural diagram of the YOLOv8 target detection model is shown in Figure 1. For a detailed introduction to the YOLOv8 target detection model, readers can refer to [41,42]. Due to space limitations, this article will not go into details.

### 2.2. Vehicle Information Feature Map (VIFM)

**Figure 2:**
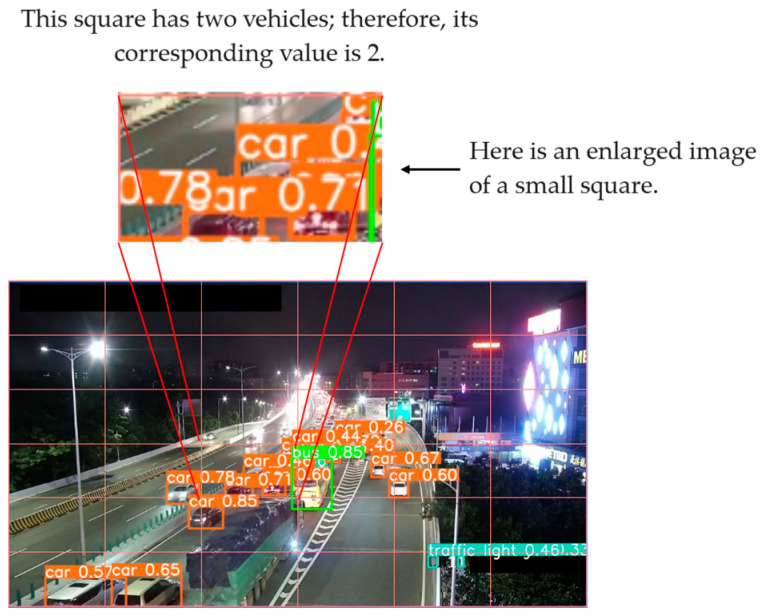
Schematic diagram of feature map extraction.

This paper proposes a vehicle information feature map (VIFM) method. After identifying the location of each car in the picture, the information needs to be processed to extract features as model input for the subsequent classifier. This article divides a two-dimensional image into m*m small squares with equal distance and then counts the number of vehicles in each small square, so that our feature map can be obtained. Its specific definition is as follows:

(1) $$f_{i,j}=\sum_{n=1}^{N}I(x_{n}\geq (i-1)\times w\;\mathrm{and}\;x_{n}<i\times w\;\mathrm{and}\;y_{n}\geq (j-1)\times h\;\mathrm{and}\;y_{n}<j\times h)$$

where *f* is the calculation result of the feature map, *N* is the number of detected vehicles, *n* is the vehicle number, *x_n_* is the *x* coordinate of the *n*-th vehicle, and *y_n_* is the *y* coordinate of the *n*-th vehicle. w and *h* are the size of a small square. *I* is an indicator function defined as follows:

(2) $$I(E)=\left\{ \begin{array}{l} 1,E=\mathrm{true}\\ 0,E=\mathrm{false} \end{array}\right.$$

where *E* is a logical expression. The schematic diagram is as Figure 2. The significance of this formula is that it first divides the image into several rectangular squares at equal distances and then counts how many vehicles are in each small square.

The detailed calculation process of the VIFM algorithm is as follows (Algorithm 1):
**Algorithm 1**: VIFMInput: *image,* Target detection *results,* Square size *m* Output: m*m feature map matrix *high* ← *image*.*shape*_0_ *width* ← *image*.*shape*_1_ *matrix* ← $0^{m\times m}$ for (*cls*, *x*, *y*, *w*, *h*) ∈ *results* do:  *center_x* ← x +$\frac{w}{2}$  *center_y* ← *y* +$\frac{h}{2}$  *index_width* ← int($\frac{center\_x}{width}$*m*)  *index_high* ← int($\frac{center\_y}{high}$*m*)   if *cls* ≠’bus’ and *cls* ≠’car’ and *cls* ≠’trunck’    continue  end if if *index_width* ≥ m or *index_high* ≥ m or *index_width* < 0 or *index_high* < 0   continue  end if  *matrix*[*index_width*,*index_high*] ← *matrix*[*index_width*,*index_high*] + 1  end for return *matrix*


After obtaining the feature map, we further extract three features from the feature map, namely the total number of feature map, the maximum number of feature map, and the number of elements greater than the threshold. The calculation is as follows:

(3) $$total=\sum_{i=1}^{m}\sum_{j=1}^{m}matrix_{i,j}$$

(4) $$max=\underset{i,j}{\max}\;matrix_{i,j}$$

(5) $$count=\sum_{i=1}^{m}\sum_{j=1}^{m}I(matrix_{i,j}>thresh)$$

where *I* is an indicator function. If the condition is true, its value is 1; otherwise it is 0. Thresh is the vehicle number threshold. In subsequent chapters, after obtaining the feature map and 3 features, this article inputs the feature map and 3 features to the classifier to identify traffic congestion.

### 2.3. Multi-Branch Convolutional Neural Network (MBCNN)

This paper proposes a multi-branch convolutional neural network (MBCNN) for automatic detection of traffic congestion. Since the features proposed in this article are two-dimensional matrix structures, their characteristics are similar to image data. Therefore, this paper uses convolutional neural network as the classifier. If the value is close to 1, it means it is congested, and if the value is close to 0, it means it is non-congested.

In order to capture the features of different sizes of the image, the image first passes through 3 convolution branches respectively. The convolution kernel sizes in the three branches are 1, 2, and 3 respectively. The padding is set to the same. That is to say, the size of the feature map is always m*m. First, in each of the three branches, the image passes through a convolutional layer, a ReLU layer, a convolutional layer, and a ReLU layer. Then, the features extracted from the three branches are concatenated together. The concatenated feature map passes through a dropout layer, a flattening layer, and finally a fully connected layer with an output length of 3 to obtain the vector representation of our image. The inference formula for the convolutional neural network part of the classifier is as follows:

(6) $$\begin{array}{l} X_{1}^{p}=W_{1,1}^{p}⊗image+b_{1,1}^{p},X_{2}^{p}=W_{1,2}^{p}⊗image+b_{1,2}^{p},\\ X_{3}^{p}=W_{1,3}^{p}⊗image+b_{1,3}^{p} \end{array}$$

(7) $$X_{1}=ReLU(X_{1}),X_{2}=ReLU(X_{2}),X_{3}=ReLU(X_{3})$$

(8) $$\begin{array}{l} X_{1}^{d}=\sum_{p=1}^{P}W_{2,1}^{p,d}⊗X_{1}^{p}+b_{2,1}^{d},X_{2}^{d}=\sum_{p=1}^{P}W_{2,2}^{p,d}⊗X_{2}^{p}+b_{2,2}^{d},\\ X_{3}^{d}=\sum_{p=1}^{P}W_{2,3}^{p,d}⊗X_{3}^{p}+b_{2,3}^{d} \end{array}$$

(9) $$X_{1}=ReLU(X_{1}),X_{2}=ReLU(X_{2}),X_{3}=ReLU(X_{3})$$

(10) $$X=X_{1}⊕X_{2}⊕X_{3}$$

*X* = *Dropout*(*X*,0.5) (11)

*X* = *Flatten*(*X*) (12)

(13) $$X=ReLu(W_{3}X+b_{3})$$

where *image* is the feature map input. ⊗ is the convolution symbol. ⊕ represents vector concatenation. *W*_1_ and *W*_2_ are the convolutional kernels of the first two convolutional layers. *b*_1_ and *b*_2_ are the bias terms of the first two convolutional layers. *ReLU* is a nonlinear activation function. *W*_3_ and *b*_3_ are parameters of the fully connected layer. The output dimension of the last fully connected layer is 3. *Dropout* is used to prevent overfitting, and its dropout probability is 0.5.

In order to consider the three features of the total number of vehicles, the maximum value, and the count value greater than the threshold, the features extracted by the convolutional neural network are first spliced with this feature. Finally, after two fully connected layers, the value is the congestion confidence. The final fully connected neural network reasoning process is as follows:

(14) X=X⊕[total,max,count]

(15) $$X=ReLU(W_{4}X+b_{4})$$

(16) $$Y=Sigmoid(W_{5}X+b_{5})$$

where ⊕ represents vector concatenation. *W*_4_ and *W*_5_ are the parameters of two fully connected layers. *b*_4_ and *b*_5_ are the bias terms of the two fully connected layers. Sigmoid is the final output layer. *Y* > 0.5 means the picture is congested, and *Y* < 0.5 means the picture is non-congested. *Total* is the total number of vehicles in the feature map, *max* is the maximum value of each element in the feature map, and *count* is the number of elements in the feature map that are greater than the threshold.

**Figure 3:**
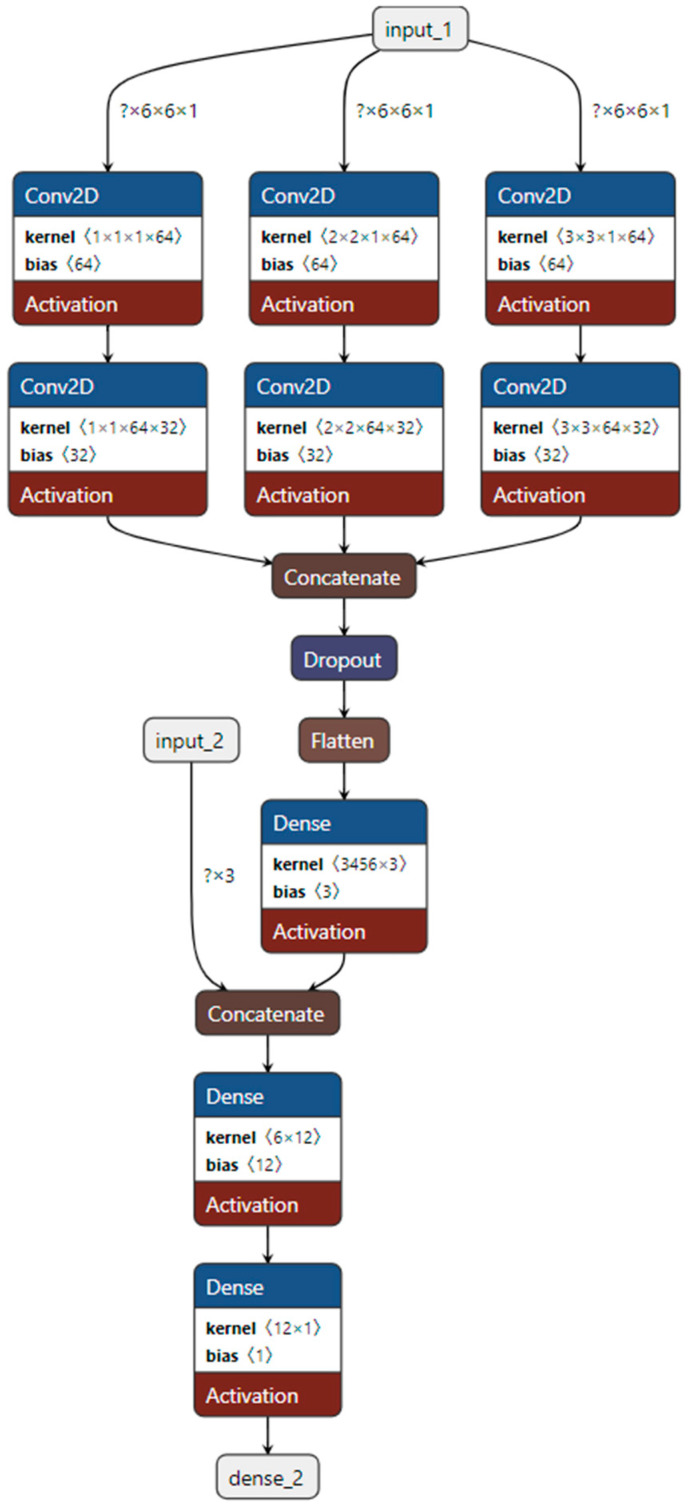
Classifier structure diagram based on convolutional neural network.

The structure of the classifier in this article is shown in the Figure 3. Among them, input1 is the feature map input, and input2 is the total number of vehicles, the maximum value and the number of elements greater than the threshold. The output is the confidence probability that there is traffic congestion in this image.

The loss function for this model training is the cross-entropy loss function, which is defined as follows:

(17) $$loss=-\frac{1}{batch\_size}\sum_{j=1}^{batch\_size}\left[y_{j}\log{\hat{y}}_{j}+(1-y_{j})\log(1-{\hat{y}}_{j})\right]$$

The classification model in this article is trained using the Adam optimization algorithm. Adam is a common optimization algorithm; readers can refer to [43].

### 2.4. Evaluation Index

This article uses F1 score as the main evaluation index for traffic congestion detection, which is as follows:

(18) $$F1=2\times \frac{precision\times recall}{precision+recall}$$

The second indicator in this article is accuracy, which is as follows:

(19) Accuracy=(TP+TN)/(TP+TN+FP+FN)

where *TP* is the true positive example, that is, the number of pictures in that actual category that are congested and are identified as congested. *TN* is the true negative example, that is, the number of pictures in that actual category that are non-congested and are recognized as non-congested. *FP* is the false positive example, that is, the number of pictures whose actual category is congestion and are recognized as non-congestion. *FN* is the false negative example, that is, the number of images in that actual category that are non-congested and are identified as congested.

## 3. Numerical Experiments

This section will conduct numerical experiments for traffic congestion detection based on actual data. Section 3.1 introduces the data used for the numerical experiments. Section 3.2 discusses the results of object detection. Section 3.3 presents the results of feature map extraction. Section 3.4 presents the classification results. Section 3.5 compares the detection accuracy of different classification models and different object detection models. Section 3.6 shows the experimental results on other datasets. Section 3.7 analyzes time and space complexity. Section 3.8 shows the choice of hyperparameter m.

### 3.1. Dataset

The Chinese City Traffic Image Database (CCTRIB) is an image dataset used for road congestion status detection [44]. CCTRIB images come from traffic videos captured by multiple city key road surveillance cameras, including surveillance videos on highways, urban roads, and expressways. The frequency of video collection is once every 500 frames. The videos collected on each key road include various situations such as lighting changes, weather changes, and different imaging scales.

**Figure 4:**
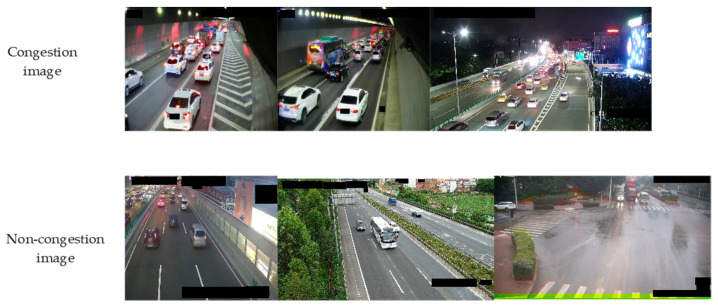
Examples of CCTRIB dataset images.

The dataset has a total of 9200 images, including 4600 traffic congestion images and 4600 non-congestion traffic images. The image resolution is between 480 × 320 and 1920 × 1080 pixels, which can be used for training and testing of road congestion detection algorithms. There are 8471 pictures in the training set and 729 pictures in the test set. The proportion of congestion and non-congestion on both the training set and the test set is 50%. Figure 4 is an example of the CCTRIB dataset. The upper part is an example of a congested image. The lower part is an example of a non-congested image.

**Figure 5:**
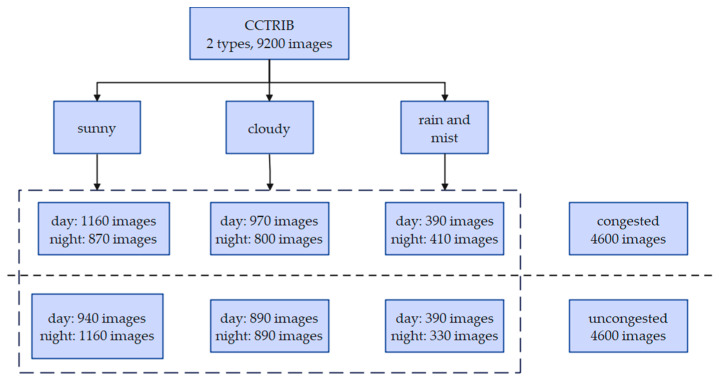
Image distribution of the CCTRIB dataset.

The congested images include 1160 daytime images and 870 nighttime images under clear skies, 970 daytime images and 800 night images under cloudy conditions, and 390 daytime images and 410 night images in rain and mist. The non-congested images include 940 daytime images and 1160 night images under clear conditions, 890 daytime images and 890 night images under cloudy conditions, 390 daytime images and 330 night images under rainy and foggy weather conditions. Figure 5 is the distribution diagram of the CCTRIB dataset images.

### 3.2. Target Detection Results

**Figure 6:**
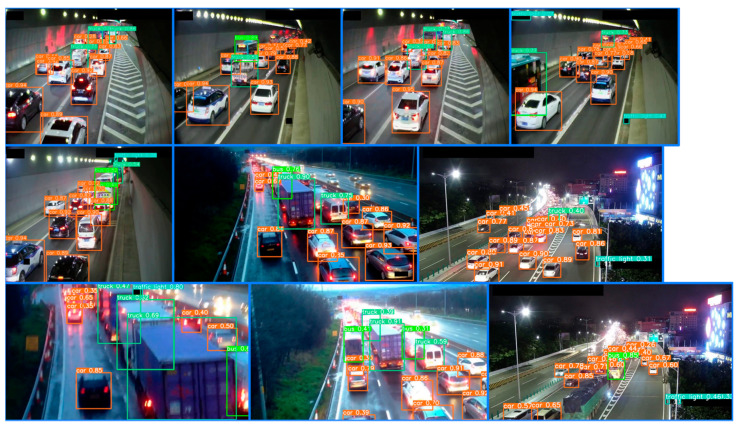
Object detection results of images from the category “congested”. Each picture in the figure is a picture from the congestion category randomly selected from the dataset.

**Figure 7:**
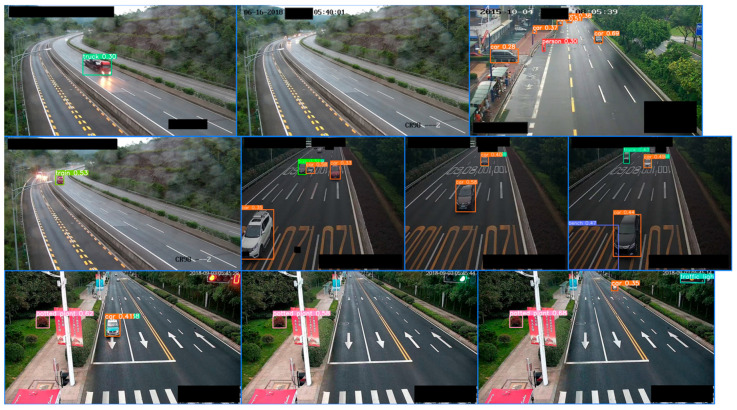
Object detection results from images with the category “non-congested”. Each picture in the figure is a non-congested picture randomly selected from the dataset.

This article first performs target detection on camera images based on the YOLOv8 model and extracts the location and quantity information of vehicles. This article detects vehicle targets in all images in the training set and test set, and plots the target locations and confidence levels. The effect of target detection on the congestion category dataset is as follows (Figure 6):

The detection effect of the target detection algorithm on non-congested datasets is as follows (Figure 7):

### 3.3. Feature Map

**Figure 8:**
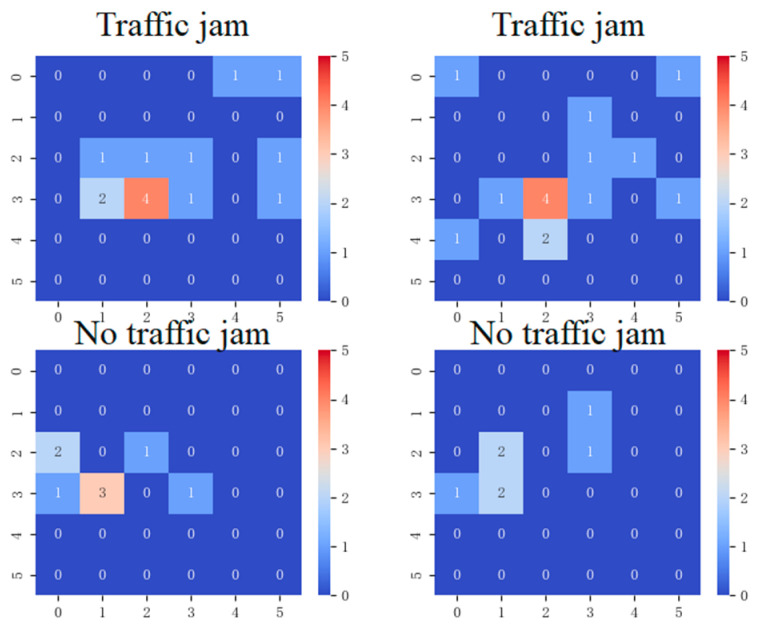
Camera image feature map extraction results.

This article extracts a feature map based on the vehicle position information obtained by the target detection model and uses it for the final classification model. We divide the entire image into m*m squares. In this experiment, we set the parameter m = 6. The reason why we determined m = 6 is that we conducted 5-fold cross-validation on the training set, and m = 6 can produce a relatively optimal result. In Section 3.8, we will discuss the value of m in detail. Then count the number of vehicles in each square. Finally, the feature map is used as the input of the classification model. Figure 8 shows the feature map extraction results of four images. The categories of the upper two images are traffic congestion, and the categories of the lower two images are non-congestion categories.

The above two pictures are feature maps of congested pictures. The following two pictures are feature maps of non-congested pictures.

### 3.4. Classification Results

We use the training set data of CCTRIB to train our MBCNN model and evaluate the accuracy of the prediction results on the test set. There are 8471 pictures in the training set and 729 pictures in the test set. The overall prediction process is to first use the pre-trained YOLOv8 model to detect vehicles, then use a VIFM to extract feature maps and advanced features, and finally use the MBCNN model to predict whether there is traffic congestion in the image.

We set the size of the feature map to 6*6 and set the element size threshold *thresh* = 3. For the first branch, the convolution kernel size is set to 1*1, and the output channel numbers of the two convolution layers are 64 and 32 respectively. The nonlinear activation function is ReLU, and the padding is set to the same. For the second branch, the convolution kernel size is set to 2*2, and the output channel numbers of the two convolution layers are 64 and 32, respectively. The nonlinear activation function is ReLU, and the padding is set to the same. For the third branch, the convolution kernel size is set to 3*3, and the channel numbers of the two convolution layers are 64 and 32, respectively. The nonlinear activation function is ReLU, and the padding is set to the same. The dropout layer has an inactivation probability of 0.5. The output size of the first fully connected layer is 3, and the activation function is ReLU. The output size of the second fully connected layer is 12, and the activation function is ReLU. The output size of the third fully connected layer is 1, and the activation function is the sigmoid function.

**Figure 9:**
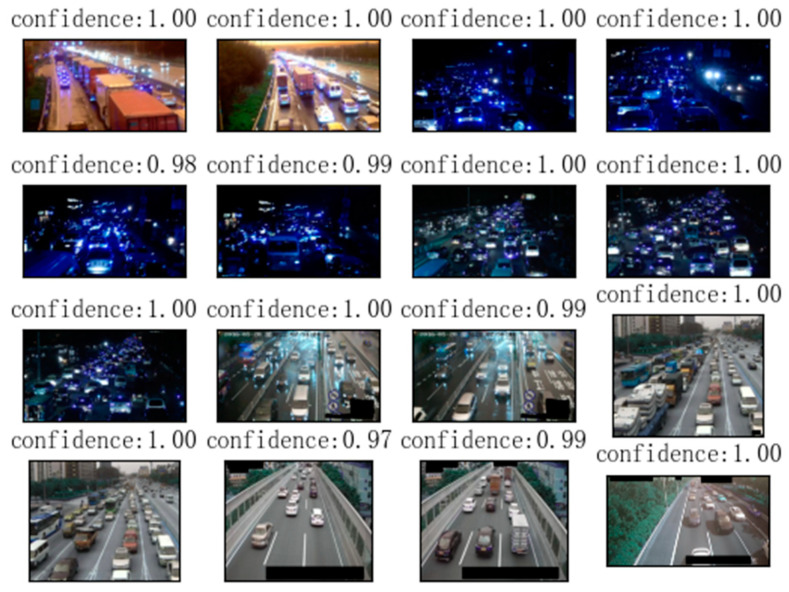
Traffic congestion prediction results. The confidence level is close to 1, which represents traffic congestion.

**Figure 10:**
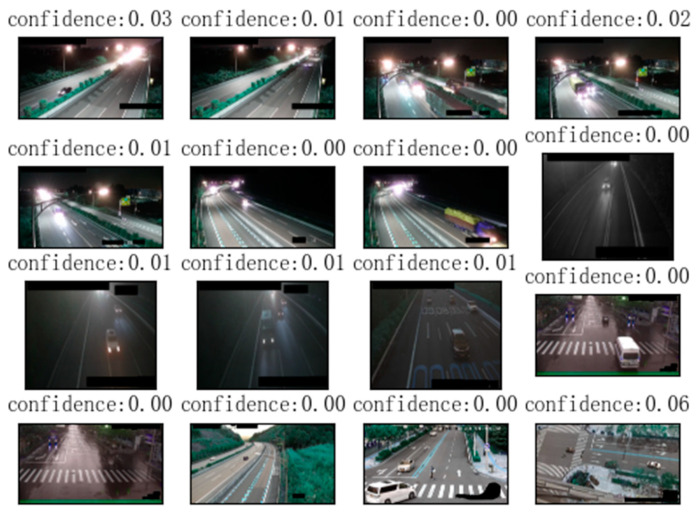
Traffic congestion prediction results. The confidence level is close to 0, which represents non-traffic congestion.

This article first shows the prediction results of some data in the test set. Figure 9 shows 16 pictures predicted to be congested. Figure 10 shows 16 pictures predicted to be non-congested. As can be seen from the figure, the method in this paper can effectively predict traffic congestion. The threshold for this experiment is 0.5. If the confidence level is greater than 0.5, it indicates a higher likelihood that the image is a congested image. Otherwise, it indicates a higher likelihood that the image is a non-congested image. In the entire test set, the F1 score of this algorithm is 98.61%, and the accuracy is 98.62%.

### 3.5. Comparative Experiment

This article uses the CNN model as the final classification model. First, our baseline model is a model that only considers the number of vehicles, that is, using YOLOv8 to detect vehicles and then counting the number in each image. Images that are larger than the threshold are identified as showing traffic congestion, while images that are smaller than the threshold are identified as showing non-congestion. The threshold is determined using a logistic regression model [45].

This article compares this model with feed-forward neural networks [46], support vector machines [47], etc. During comparison, the feature map is first converted into a 1-dimensional vector and then input into the classification model for classification.

The number of hidden layer units of the feed-forward neural network is set to 256, the hidden layer activation function is set to the ReLU function, the number of output layer units is 1, the activation function is the sigmoid function, the binary cross-entropy loss function is used, and the optimization algorithm uses the Adam algorithm. The parameter *C* of the SVM classifier is set to 1, the kernel function is the rbf function, the order is set to 3, the minimum tolerated accuracy is set to 0.001, the block size is set to 200, and the decision function is ovr.

At the same time, this article also compares the classification accuracy of several large-scale deep neural networks, as shown in Table 1. We utilize a large pre-trained network to extract features directly on the original images. For the VGG16 model [48], the output is a 512-dimensional feature vector. For the Resnet50 [49] model, the output is a 2048-dimensional feature vector. For the EfficientNet b7 [50] model, the output is a 2560-dimensional feature vector. For large pre-trained networks, the final classifier settings are thus: the number of hidden layer units is 128, the activation function is the ReLU function, the number of output layer units is 1, and the activation function is the sigmoid function. It is worth noting that it is still relatively rare to use convolutional neural networks for traffic congestion detection. The convolutional neural network references cited in this article have applications in other fields. These models are mainly used for comparative studies.

In order to choose the optimal target detection model, this article also compares the traffic congestion detection results obtained when using YOLOv8 and YOLOv5 [20,21], SSD [22,23] and the Haar Cascade models [19] (Table 2).

**Figure 11:**
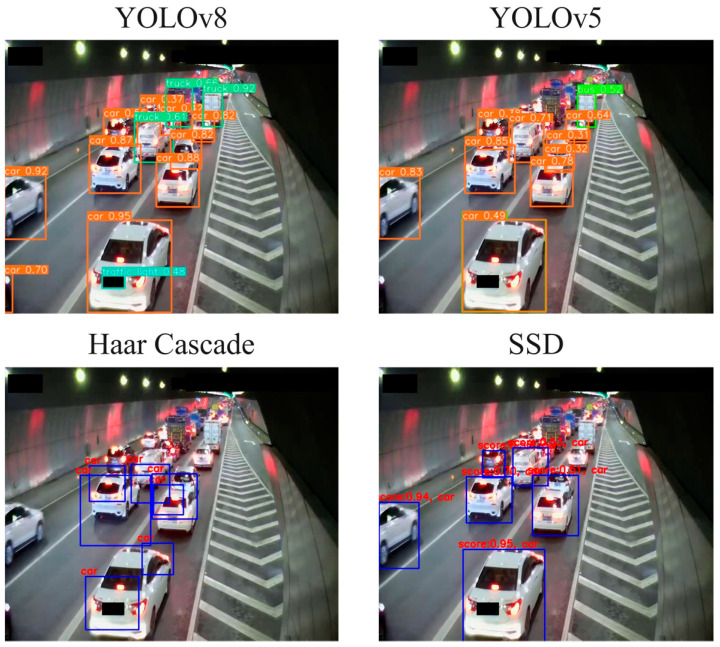
Detection results of four target detection models.

In order to more intuitively display the detection effects of the four target detection models, we draw the detection results of a picture in the dataset, and the results are in Figure 11. It can be seen that the YOLOv8 and YOLOv5 models achieve relatively better results.

### 3.6. Experiments on Other Datasets

To further verify the generalization performance of the proposed method, we conducted inference experiments on a subset of data from the traffic net dataset [51]. The traffic net dataset has a total of four categories of data, including accidents, dense traffic, fires, and sparse traffic. Each category has 1100 images.

We trained our model based on the data of dense traffic and sparse traffic categories in the traffic net dataset and validate the effectiveness of various methods on the test set. The hyperparameter calibration of these models is the same as that in Section 3.5. The results are shown in Table 3. It can be seen that the model in this paper achieves better results than the baseline model, machine learning model, and pre-trained convolutional neural network model. It is worth noting that due to the different installation locations of some cameras in the traffic net dataset compared to CCTRIB, and because the training set of the traffic-net dataset is only 1800 images, the generalization performance is slightly worse, which is normal.

**Figure 12:**
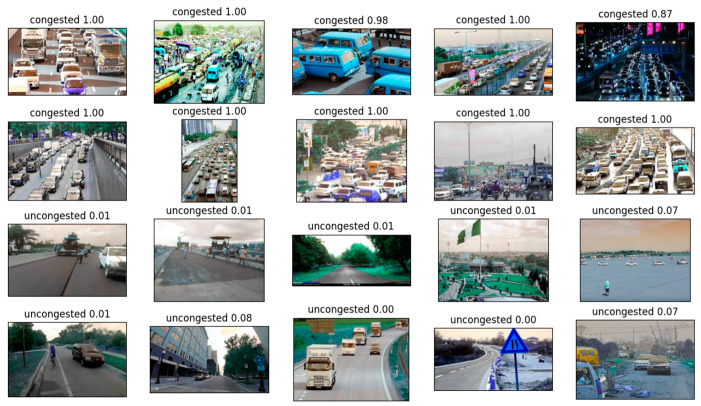
The inference results of the method on the traffic net dataset. The text in each image is the prediction category, and the number is the prediction confidence.

We extracted 10 images each from the dense and sparse traffic categories, for a total of 20 images, and used the MBCNN model for inference. The results are as follows (Figure 12):

As can be seen from the figure, the model in this article can effectively identify congested and non-congested images in the traffic net dataset.

### 3.7. Time and Space Complexity Analysis

In order to more comprehensively analyze the performance of this algorithm, this article compares the time complexity and space complexity of the algorithm. The main frequency of our computer is 1.9 HZ. The memory size is 16 GB, and the operating system is 64-bit. The analysis results are shown in Table 4. It can be seen that the time and space complexity of this method can well meet the needs of the current computing power.

### 3.8. The Influence of Hyperparameter m on the Experimental Results

To determine the impact of the feature map size m on the experimental classification accuracy, we used a 5-fold cross-validation method with a dataset. That is, 20% of the data from the training set were used as the validation set. This article tested the classification performance on the validation set when m was 4, 5, 6, and 7. The results are shown in the following table. From Table 5, it can be seen that the optimal value of m is 6.

## 4. Conclusions

This article studies an automatic detection method of traffic congestion based on image data and proposes a new traffic congestion feature extraction method VIFM and a new traffic congestion classifier MBCNN, which can provide effective data support for traffic management, reduce system operating costs, and provide new methods for automatic detection.

This method is based on the YOLOv8 model to detect vehicle information in images. Based on the VIFM method, it extracts feature maps and high-level features of vehicle information. This method uses MBCNN to identify whether the image is a traffic congestion image. The method proposed in this paper can effectively utilize the existing massive network of cameras in the traffic system to automatically detect traffic congestion without increasing hardware costs.

In Section 3, the following are discussed. (1) This article analyzes the detection results of YOLOv8. (2) This article analyzes the results of VIFM extracting feature maps. (3) This article analyzes the results of MBCNN for congestion recognition. (4) This article compares the congestion recognition results of different classifiers and different object detection models. (5) This article analyzes the spatial and temporal complexity of our method.

This article verifies the effectiveness of the proposed method on the CCTRIB dataset. (1) This article verifies the effectiveness of the VIFM method and the MBCNN classifier. The classification model in this article achieves an F1 score of 98.61% and an accuracy of 98.62% on the test set. (2) This article compares the detection accuracy of this model with other object detection models, other convolutional neural network models, other deep learning models, and baseline models (see Table 1, Table 2 and Table 3). Numerical experiments show that the proposed method achieves good results.

In future research, we will study end-to-end multi-stage traffic congestion recognition methods which integrate target detection, feature map extraction, and congestion classification into an end-to-end model to further improve the accuracy and computational efficiency of traffic congestion recognition.

## Figures and Tables

**Table 1:** Comparison between the classification model of this article and other classification models.

Model	F1 (%)	Accuracy (%)
Baseline [45]	85.23	85.45
MBCNN (ours)	**98.61**	**98.62**
FNN [46]	97.04	97.11
SVM [47]	98.17	97.80
CNN(VGG16) [48]	98.12	98.08
CNN(Resnet50) [49]	97.17	97.11
CNN(EfficientNe_b7) [50]	95.25	95.19

**Table 2:** Comparison of target detection models.

	F1 (%)	Accuracy (%)
Haar Cascade [19]	71.54	75.44
YOLOv8 [20]	98.61	98.62
YOLOv5 [21]	95.38	95.47
SSD [22]	72.49	76.26

**Table 3:** Comparison of prediction results on the traffic net dataset.

Model	F1 (%)	Accuracy (%)
Baseline	75.68	77.5
MBCNN (ours)	**85.38**	87.25
FNN	83.05	85.25
SVM	82.35	85
CNN(VGG16)	83.74	83.25
CNN(Resnet 50)	79.09	79.25
CNN(EfficientNe_b7)	77.51	73.75

**Table 4:** Time and space complexity analysis results.

Attributes	Average Value
YOLOv8 inference time	790 ms
Feature map extraction time	negligible
Classifier inference time	0.13 ms
Object detection model size	133 MB
Classification model size	62 KB

**Table 5:** Results of different hyperparameters m.

m Value	F1 (%)	Accuracy (%)
4	92.15	95.91
**5**	**98.17**	**98.07**
6	98.29	98.64
7	94.33	97.08

## Data Availability

The data that support the findings of this study are available from OpenITS Open Data, but restrictions apply to the availability of these data, which were used under license for the current study, and so the data are not publicly available. Data are however available from the authors upon reasonable request and with the permission of OpenITS Open Data.

## References

[1] Kumar N., Raubal M. (2021). Applications of deep learning in congestion detection, prediction and alleviation: A survey. Transp. Res. Part C Emerg. Technol..

[2] Li Z., Yu H., Zhang G., Dong S., Xu C.Z. (2021). Network-wide traffic signal control optimization using a multi-agent deep reinforcement learning. Transp. Res. Part C Emerg. Technol..

[3] Jiang S., Feng Y., Liao X., Wu H., Liu J., Onasanya B.O. (2024). A Novel Spatiotemporal Periodic Polynomial Model for Predicting Road Traffic Speed. Symmetry.

[4] Sadollah A., Gao K., Zhang Y., Zhang Y., Su R. (2019). Management of traffic congestion in adaptive traffic signals using a novel classification-based approach. Eng. Optim..

[5] Jeng S.T., Chu L. A high-definition traffic performance monitoring system with the inductive loop detector signature technology. Proceedings of the 17th International IEEE Conference on Intelligent Transportation Systems (ITSC).

[6] Tasgaonkar P.P., Garg R.D., Garg P.K. (2020). Vehicle detection and traffic estimation with sensors technologies for intelligent transportation systems. Sens. Imaging.

[7] Tong L., Li Z. (2014). Study on the road traffic survey system based on micro-ferromagnetic induction coil sensor. Sens. Transducers.

[8] Thianniwet T., Phosaard S., Pattara-Atikom W. (2009). Classification of road traffic congestion levels from GPS data using a decision tree algorithm and sliding windows. Proc. World Congr. Eng..

[9] Yong-chuan Z., Xiao-qing Z., Zhen-ting C. (2011). Traffic congestion detection based on GPS floating-car data. Procedia Eng..

[10] Keskin M., Akkamis M., Sekerli Y.E. An overview of GNSS and GPS based velocity measurement in comparison to other techniques. Proceedings of the International Conference on Energy Research.

[11] Cui H., Yuan G., Liu N., Xu M., Song H. (2020). Convolutional neural network for recognizing highway traffic congestion. J. Intell. Transp. Syst..

[12] Calderoni L., Maio D., Rovis S. (2014). Deploying a network of smart cameras for traffic monitoring on a “city kernel”. Expert Syst. Appl..

[13] Iftikhar S., Asim M., Zhang Z., Muthanna A., Chen J., El-Affendi M., Abd El-Latif A.A. (2023). Target detection and recognition for traffic congestion in smart cities using deep learning-enabled UAVs: A review and analysis. Appl. Sci..

[14] Liu X., Gao W., Feng D., Gao X. Abnormal traffic congestion recognition based on video analysis. Proceedings of the 2020 IEEE Conference on Multimedia Information Processing and Retrieval (MIPR).

[15] Soo S. (2014). Object Detection Using Haar-Cascade Classifier.

[16] Saeedmanesh M., Kouvelas A., Geroliminis N. (2021). An extended Kalman filter approach for real-time state estimation in multi-region MFD urban networks. Transp. Res. Part C Emerg. Technol..

[17] Chakraborty P., Adu-Gyamfi Y.O., Poddar S., Ahsani V., Sharma A., Sarkar S. (2018). Traffic congestion detection from camera images using deep convolution neural networks. Transp. Res. Rec..

[18] Lam C.T., Gao H., Ng B. A real-time traffic congestion detection system using on-line images. Proceedings of the 2017 IEEE 17th International Conference on Communication Technology (ICCT).

[19] Aarthi V. Prototype Design of Intelligent Traffic Signal Control using Haar Cascade Classifier. Proceedings of the 2021 Sixth International Conference on Wireless Communications, Signal Processing and Networking (WiSPNET).

[20] Lin J.P., Sun M.T. A YOLO-based traffic counting system. Proceedings of the 2018 Conference on Technologies and Applications of Artificial Intelligence (TAAI).

[21] Al-qaness M.A., Abbasi A.A., Fan H., Ibrahim R.A., Alsamhi S.H., Hawbani A. (2021). An improved YOLO-based road traffic monitoring system. Computing.

[22] Biswas D., Su H., Wang C., Stevanovic A., Wang W. (2019). An automatic traffic density estimation using Single Shot Detection (SSD) and MobileNet-SSD. Phys. Chem. Earth Parts A/B/C.

[23] You S., Bi Q., Ji Y., Liu S., Feng Y., Wu F. (2020). Traffic sign detection method based on improved SSD. Information.

[24] Zuo Z., Yu K., Zhou Q., Wang X., Li T. Traffic signs detection based on faster r-cnn. Proceedings of the 2017 IEEE 37th International Conference on Distributed Computing Systems Workshops (ICDCSW).

[25] Luo J.Q., Fang H.S., Shao F.M., Zhong Y., Hua X. (2021). Multi-scale traffic vehicle detection based on faster R–CNN with NAS optimization and feature enrichment. Def. Technol..

[26] Nguyen H.N., Krishnakumari P., Vu H.L., Van Lint H. Traffic congestion pattern classification using multi-class svm. Proceedings of the 2016 IEEE 19th International Conference on Intelligent Transportation Systems (ITSC).

[27] Khalifa A.B., Alouani I., Mahjoub M.A., Amara N.E.B. (2020). Pedestrian detection using a moving camera: A novel framework for foreground detection. Cogn. Syst. Res..

[28] Wu B., Nevatia R. Detection of multiple, partially occluded humans in a single image by bayesian combination of edgelet part detectors. Proceedings of the Tenth IEEE International Conference on Computer Vision (ICCV’05).

[29] Xie Z., Yang R., Guan W., Niu J., Wang Y. A Novel Descriptor for Pedestrian Detection Based on Multi-layer Feature Fusion. Proceedings of the 2020 IEEE International Conference on Real-Time Computing and Robotics (RCAR).

[30] Patel C.I., Labana D., Pandya S., Modi K., Ghayvat H., Awais M. (2020). Histogram of oriented gradient-based fusion of features for human action recognition in action video sequences. Sensors.

[31] Wali S.B., Abdullah M.A., Hannan M.A., Hussain A., Samad S.A., Ker P.J., Mansor M.B. (2019). Vision-based traffic sign detection and recognition systems: Current trends and challenges. Sensors.

[32] Singh I., Singh S.K., Kumar S., Aggarwal K. (2022). Dropout-VGG based convolutional neural network for traffic sign categorization. Congress on Intelligent Systems: Proceedings of CIS.

[33] Boudissa M., Kawanaka H., Wakabayashi T. Traffic Landmark Quality Evaluation Using Efficient VGG-16 model. Proceedings of the 2022 Joint 12th International Conference on Soft Computing and Intelligent Systems and 23rd International Symposium on Advanced Intelligent Systems (SCIS&ISIS).

[34] Prawinsankar D., Gunasekaran M., Gopalakrishnan B., Purusothaman P. Traffic Congession Detection through Modified Resnet50 and Prediction of Traffic using Clustering. Proceedings of the 2021 Smart Technologies, Communication and Robotics (STCR).

[35] Wang Y., Zhao Z., He J., Zhu Y., Wei X. A method of vehicle flow training and detection based on ResNet50 with CenterNet method. Proceedings of the 2021 International Conference on Communications, Information System and Computer Engineering (CISCE).

[36] Zhang S., Bu Y., Chen B., Lu X. Transfer learning for encrypted malicious traffic detection based on efficientnet. Proceedings of the 2021 3rd International Conference on Advances in Computer Technology, Information Science and Communication (CTISC).

[37] Koonce B., Koonce B.E. (2021). Convolutional Neural Networks with Swift for Tensorflow: Image Recognition and Dataset Categorization.

[38] Kheder M.Q., Mohammed A.A. (2023). Transfer Learning Based Traffic Light Detection and Recognition Using CNN Inception-V3 Model. Iraqi J. Sci..

[39] Lin C., Li L., Luo W., Wang K.C., Guo J. (2019). Transfer learning based traffic sign recognition using inception-v3 model. Periodica Polytechnica Transp. Eng..

[40] Impedovo D., Balducci F., Dentamaro V., Pirlo G. (2019). Vehicular traffic congestion classification by visual features and deep learning approaches: A comparison. Sensors.

[41] Talaat F.M., ZainEldin H. (2023). An improved fire detection approach based on YOLO-v8 for smart cities. Neural Comput. Appl..

[42] Sohan M., Sai Ram T., Reddy R., Venkata C. (2024). A Review on YOLOv8 and Its Advancements. Proceedings of the International Conference on Data Intelligence and Cognitive Informatics.

[43] LeCun Y., Bengio Y., Hinton G. (2015). Deep learning. Nature.

[44] Li X., OpenITS Org (2022). OpenData V15.0-Chinese City Traffic Image Database (CCTRIB). http://www.openits.cn/openData4/824.jhtml.

[45] LaValley M.P. (2018). Logistic regression. Circulation.

[46] Aldakheel F., Satari R., Wriggers P. (2021). Feed-forward neural networks for failure mechanics problems. Appl. Sci..

[47] Xu L., Wang X., Bai L., Xiao J., Liu Q., Chen E., Jiang X., Luo B. (2020). Probabilistic SVM classifier ensemble selection based on GMDH-type neural network. Pattern Recognit..

[48] Theckedath D., Sedamkar R.R. (2020). Detecting affect states using VGG16, ResNet50 and SE-ResNet50 networks. SN Comput. Sci..

[49] Hossain M.B., Iqbal S.H.S., Islam M.M., Akhtar M.N., Sarker I.H. (2022). Transfer learning with fine-tuned deep CNN ResNet50 model for classifying COVID-19 from chest X-ray images. Inform. Med. Unlocked.

[50] Reza A.W., Hasan M.M., Nowrin N., Shibly M.A. (2021). Pre-trained deep learning models in automatic COVID-19 diagnosis. Indones. J. Electr. Eng. Comput. Sci..

[51] Olafenwa M. Traffic Net Dataset. https://github.com/OlafenwaMoses/Traffic-Net.

